# A Novel Partial EMT-Associated Transcriptomic Signature for Prognostic Stratification in Ovarian Cancer

**DOI:** 10.32604/or.2026.074383

**Published:** 2026-04-22

**Authors:** Chia-Chia Chao, Cheng-Yao Lin, Po-Chun Chen, Wen-Tsung Huang, Teng-Song Weng, Sheng-Yen Hsiao

**Affiliations:** 1 Department of Respiratory Therapy, Fu Jen Catholic University, New Taipei City, Taiwan; 2 Division of Hematology-Oncology, Department of Internal Medicine, Chi Mei Medical Center, Liouying, Tainan, Taiwan; 3 Department of Senior Welfare and Services, Southern Taiwan University of Science and Technology, Tainan, Taiwan; 4 Department of Environmental and Occupational Health, College of Medicine, National Cheng Kung University, Tainan, Taiwan; 5 School of Life Science, National Taiwan Normal University, Taipei, Taiwan; 6 Translational Medicine Center, Shin-Kong Wu Ho-Su Memorial Hospital, Taipei, Taiwan; 7 Department of Medical Research, China Medical University Hospital, China Medical University, Taichung, Taiwan; 8 Department of Pharmacy, Chi Mei Medical Center, Liouying, Tainan, Taiwan; 9 Department of Nursing, Chung Hwa University of Medical Technology, Tainan, Taiwan

**Keywords:** Partial epithelial-mesenchymal transition, ovarian cancer, prognosis, transcriptomic risk score, partial epithelial–mesenchymal transition (p-EMT) signature

## Abstract

**Background:**

Partial epithelial–mesenchymal transition (p-EMT) is a dynamic cellular state associated with metastasis and adverse outcomes in multiple cancers, but its prognostic significance in ovarian cancer remains unclear. This study aimed to develop and validate an ovarian cancer–specific transcriptomic signature based on p-EMT–related genes, and to determine whether this signature can improve prognostic stratification and overall survival prediction across independent cohorts.

**Methods:**

A pan-cancer p-EMT gene set was curated from ten published studies. Using transcriptomic and clinical data from TCGA-OV (n = 488), a six-gene p-EMT signature was developed via LASSO regression to generate a patient-specific risk score. The score was integrated with clinical variables to construct a prognostic nomogram and validated in the external GEO cohort GSE140082 (n = 380) and GSE165808 (n = 51).

**Results:**

A six-gene p-EMT transcriptomic signature (ADAM9, ANXA8L1, FSTL3, RABAC1, TPM4, and TWIST1) was significantly associated with overall survival (OS) and stratified patients into high- and low-risk groups (adjusted HR = 1.74, *p* < 0.001). Incorporation with age and FIGO stage in a nomogram improved predictive performance, with AUCs of 0.727, 0.700, and 0.656 at 1-, 3-, and 5-year OS, respectively. External validation in GSE140082 and GSE165808 confirmed model robustness, yielding 3-year AUCs of 0.630 and 0.826, respectively, demonstrating preserved prognostic value across independent cohorts and disease stages.

**Conclusions:**

This six-gene p-EMT transcriptomic signature demonstrates prognostic value in ovarian cancer and offers potential for individualized risk stratification and clinical decisionsupport.

## 1 Introduction

Ovarian cancer ranks as the eighth most common malignancy in women, contributing to 3.4% of new cancer diagnoses and 4.8% of female cancer-related deaths worldwide in 2022 [1]. Although global survival has gradually improved [2], outcomes for patients presenting with advanced-stage disease remain poor, underscoring the need for improved tools for early detection and prognostic evaluation [3]. Because symptoms are typically nonspecific, only a small proportion of patients—approximately 15%—are diagnosed at stage I, while most present with advanced disease, for which the 5-year overall survival (OS) is near 30% [4,5]. Even with standard cytoreductive surgery and platinum-based chemotherapy, 70%–90% of patients with advanced disease experience recurrence within 18 months [6]. These clinical realities highlight the limitations of current markers such as CA125 and HE4, which offer only modest prognostic discrimination [7], and emphasize the need for molecular signatures that can better capture tumor biology and refine risk stratification.

Epithelial–mesenchymal transition (EMT) is a key process in cancer progression, involving the loss of epithelial characteristics and acquisition of mesenchymal traits [8]. Increasing evidence indicates that this transition is not strictly binary. Instead, cancer cells frequently occupy intermediate, plastic states collectively described as partial EMT (p-EMT), in which epithelial adhesion features coexist with mesenchymal motility programs [9]. These hybrid states confer enhanced invasiveness, metastatic capability, and resistance to therapy [10]. Numerous p-EMT–associated genes and transcriptional programs have been linked to tumor aggressiveness and adverse survival across cancer types [11–13]. Recent analyses in head and neck, breast, and lung cancers further demonstrate that p-EMT signatures may serve as meaningful prognostic or predictive biomarkers [14–16]. However, whether p-EMT holds similar prognostic relevance in ovarian cancer has not been systematically examined. Existing studies have largely centered on traditional EMT markers, providing limited insight into the biological and clinical impact of hybrid epithelial/mesenchymal states.

To fill this gap, we sought to determine whether transcriptomic features of partial EMT could provide clinically meaningful prognostic information in ovarian cancer. Based on evidence from multiple tumor types, we hypothesized that a p-EMT–associated transcriptional program may identify biologically aggressive disease and improve risk stratification beyond conventional clinicopathologic factors. Our aim was to improve individualized prognostic assessment and support the development of biologically informed stratification tools for ovarian cancer.

## 2 Methods

### 2.1 Curation of Pan-Cancer p-EMT Gene Set from Published Studies

A pan-cancer p-EMT gene set was constructed through a literature review of studies reporting p-EMT-associated gene expression in multiple solid tumors, including head and neck, oral cavity, lung, liver, and breast cancers [11–13,17–23]. The search was performed in PubMed (https://pubmed.ncbi.nlm.nih.gov/), restricted to the past 10 years, using the keywords “partial epithelial–mesenchymal transition,” “partial EMT,” “p-EMT,” “hybrid EMT,” and “intermediate EMT state.” Both original studies and relevant review articles were considered if they described transcriptomic or gene-signature features related to partial or hybrid EMT states in human cancers. Studies that focused solely on canonical EMT markers or reported only single-gene mechanistic findings without broader transcriptomic evidence were excluded. Gene lists extracted from eligible publications were combined, deduplicated, and standardized according to HUGO gene nomenclature. No weighting or confidence criteria were applied during gene integration, as the aim was to construct an inclusive pan-cancer p-EMT candidate gene set. All genes reported by eligible studies were therefore incorporated. This process yielded a unified set of 140 p-EMT–related genes that served as the candidate pool for subsequent prognostic modeling.

### 2.2 Data Acquisition and Preprocessing from the TCGA-OV Datasets

Clinical and transcriptomic data for ovarian serous cystadenocarcinoma (TCGA-OV) patients were obtained directly from the Genomic Data Commons (GDC) data portal via the browser interface (https://portal.gdc.cancer.gov/) on 21 May 2025, using the harmonized GRCh38 release. The initial cohort comprised 587 patients classified as “primary tumor.” To ensure cohort homogeneity, three patients with synchronous primary tumors were excluded, yielding 584 primary ovarian cancer cases. We further restricted the dataset to patients with a primary site code of “C56.9” (ovary), resulting in 577 eligible cases. Clinical records with missing key variables—including mortality status (n = 2), FIGO stage (n = 3), tumor grade (n = 13), or race information (n = 27)—were excluded, leaving 532 patients for clinical analyses.

For transcriptomic analyses, raw CEL files downloaded from the GDC were used. Microarray data were normalized using the Robust Multi-Array Average (RMA) algorithm implemented in the *affy* (v1.86.0) package in R. Probe sets were mapped to gene symbols using the *hthgu133a.db* (v3.13.0) annotation R package. After excluding cases without matched expression profiles, 488 patients with complete clinical and transcriptomic information constituted the final analytic cohort for p-EMT risk modeling. Clinical covariates incorporated into the prognostic model included age, FIGO stage, tumor grade, and race.

### 2.3 Development of the p-EMT Risk Score

RMA-normalized gene expression values were used for survival modeling. Univariate Cox regression was first performed to evaluate the association between OS and the expression of each of the 140 predefined p-EMT–related genes. This analysis served as an initial feature-screening step to reduce dimensionality rather than for formal hypothesis testing; therefore, a nominal *p*-value threshold (*p* < 0.05) was applied without multiple-testing correction. Genes passing this initial screen were subsequently entered into a least absolute shrinkage and selection operator (LASSO) Cox regression model using the *glmnet* (v4.1-10) package in R. Ten-fold cross-validation was employed to determine the optimal penalty parameter for final feature selection. A patient-specific p-EMT risk score was computed as the weighted sum of RMA-normalized gene expression values of the selected genes, using their corresponding LASSO-derived coefficients:

$$\text{p}-\text{EMT risk score }=\sum_{i=1}^{n}(Coeffi\cdot Expri)$$

where *Expri* denotes the normalized expression of gene *i*, and *Coeffi* represents the corresponding LASSO coefficient.

### 2.4 Survival Analysis and Risk Stratification

Categorical variables were summarized as counts and percentages and compared between survivors and non-survivors using the Chi-square or Fisher’s exact test, as appropriate. Continuous variables with non-normal distributions were reported as medians with interquartile ranges (IQRs) and compared using the Wilcoxon rank-sum test. Normal distribution examination was using Shapiro-Wilk test. OS was estimated using the Kaplan–Meier method, and differences between groups were assessed with the log-rank test. The prognostic significance of the p-EMT risk score was evaluated using Cox proportional hazards regression models. The risk score was analyzed both as a continuous variable and as a dichotomized variable. Several stratification strategies were explored—including median-based, optimal cut-point, and percentile-based thresholds—and the 65th percentile cut-off was ultimately selected because it yielded the highest concordance index (C-index) in the training cohort. Variables with *p* < 0.05 in univariate analysis were included in the multivariate Cox model to adjust for potential confounders. Hazard ratios (HRs) and 95% confidence intervals (CIs) were calculated to quantify associations with OS. The PH assumption was evaluated using Schoenfeld residual–based diagnostics with resampling, including the supremum test for proportional hazards. The assumption was generally satisfied for the p-EMT risk score and tumor stage, whereas age showed evidence of non-proportional hazards. Because the primary objective of this study was risk prediction, age was retained in the model. A sensitivity analysis allowing a time-varying effect for age was conducted by including an interaction term between age (per 1-year increase) and log(time), with time measured in months. All statistical tests were two-sided, and a *p* < 0.05 was considered statistically significant. Data management and statistical analyses are conducted with SAS version 9.4 software (SAS Institute, Inc.).

### 2.5 Construction and Evaluation of the Prognostic Nomogram

A prognostic nomogram was constructed based on the multivariate Cox proportional hazards model to quantify individualized OS probabilities by incorporating the p-EMT risk score with independent clinical predictors. The nomogram was designed to estimate 1-, 3-, and 5-year OS in ovarian cancer patients. Model development was performed using the TCGA-OV cohort, and external validation was conducted in two independent datasets. GSE140082 (n = 380) is derived from the ICON7 phase III clinical trial and profiled using Illumina DASL arrays; survival endpoints follow trial definitions for OS (randomization to death) and PFS (randomization to progression or death). GSE165808 (n = 51) is an RNA-seq dataset of primary high-grade serous ovarian carcinoma with OS available using standard clinical definitions. Discrimination was assessed using time-dependent ROC analysis, with operating characteristic curve (AUC) at 1-, 3-, and 5-year OS. Time-dependent AUC reflects the model’s ability to distinguish survival outcomes at specific time points, whereas Harrell’s concordance index (C-index) provides a global measure of concordance between predicted and observed survival over the entire follow-up period.

Calibration was evaluated with calibration plots comparing predicted vs. observed survival probabilities. Kaplan–Meier survival curves with log-rank tests were used to illustrate and compare OS differences between patients stratified into high- and low-risk groups based on the p-EMT risk score, providing complementary clinical interpretability to numerical metrics of discrimination. The risk-score cut-off was selected by comparing several candidate thresholds (median, optimal cut-point, and percentile-based values), with the 65th percentile chosen for primary analyses because it achieved the highest C-index.

Statistical analyses were conducted using R version 4.4.1 (http://www.r-project.org/) with the *survival* (v3.8-3), *rms* (v6.8-1), *timeROC* (v0.4), *ggplot2* (v4.0.0), *survminer* (v0.5.0). Specifically, the *survival* package was used for Cox proportional hazards modeling; *rms* supported nomogram construction and calibration; *timeROC* was used for time-dependent ROC and AUC estimation; and *survminer* together with *ggplot2* was used for visualization of survival curves and graphical outputs.

### 2.6 External Validation Using the GSE140082 and GSE165808 Cohorts

The prognostic model, integrating the p-EMT risk score with clinical variables (age and FIGO stage), was applied to an independent external cohorts (GSE140082, n = 380; GSE165808, n = 51) to assess its generalizability [24,25]. Gene expression and corresponding clinical data were retrieved directly from the Gene Expression Omnibus (GEO) database (https://www.ncbi.nlm.nih.gov/geo/query/acc.cgi), with GSE140082 retrieved on 05 June 2025 and GSE165808 retrieved on 17 November 2025. For consistency with the TCGA-OV training cohort, p-EMT risk scores were calculated using the same formula and LASSO-derived coefficients. Patients were then stratified into high- and low-risk groups based on the 65th percentile of the risk score distribution within each dataset. To account for cross-platform differences between RNA-seq (TCGA-OV) and microarray-based GEO datasets, a dataset-specific percentile cutoff strategy was used, which follows established external-validation principles and minimizes platform-related bias while maintaining consistency with the training cohort [26]. Prognostic performance in the external cohorts was evaluated using time-dependent ROC curves and AUC values at 1-, 3-, and 5-year OS, Harrell’s concordance index (C-index), Kaplan–Meier survival analysis with log-rank testing, and calibration curves to assess agreement between predicted and observed survival probabilities. This external validation framework was designed to rigorously evaluate the reproducibility and generalizability of the p-EMT-based prognostic model across independent transcriptomic platforms.

### 2.7 Statistical Analysis

Associations between clinical or molecular variables and OS were assessed using univariate and multivariate Cox proportional hazards regression models with the survival package. Prognostically relevant genes were selected by LASSO regression using the glmnet package. Survival differences between high- and low-risk groups were evaluated by Kaplan–Meier analysis with log-rank tests. Model discrimination was quantified using time-dependent receiver operating characteristic (ROC) curves generated with the timeROC package, and a prognostic nomogram with calibration curves was constructed using the rms package. Additional visualization and analysis were performed using the survminer, pROC, and ggplot2 packages. Two-sided *p* values < 0.05 were considered statistically significant unless otherwise specified.

## 3 Results

### 3.1 Curation and Transcriptomic Profiling of Pan-Cancer p-EMT Genes

**Figure 1:**
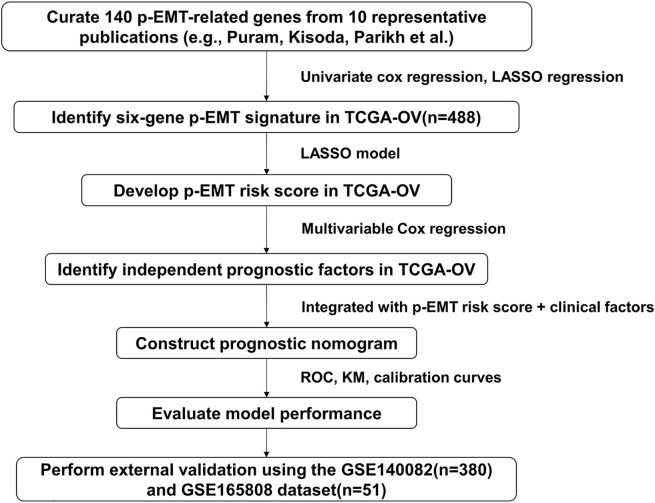
Workflow for development and validation of the partial epithelial–mesenchymal transition (p-EMT)–based prognostic model. A total of 140 p-EMT–associated genes were curated from ten published studies. Univariate Cox and LASSO regression analyses were conducted using the TCGA ovarian cancer cohort (TCGA-OV, n = 488) to identify six prognostically relevant genes. These were used to construct a p-EMT risk score, which was integrated with clinical variables to build a multivariable prognostic nomogram. Model performance was evaluated through receiver operating characteristic (ROC) curves, Kaplan–Meier survival analysis, and calibration plots, and subsequently validated in an independent cohort (GSE140082, n = 380; GSE165808, n = 51).

Fig. 1 provides an overview of the study workflow, including the development and external validation of a prognostic model for ovarian cancer. To examine whether p-EMT–associated transcriptomic features could inform prognostic stratification in ovarian cancer, we first curated a pan-cancer p-EMT gene set from ten published studies, resulting in 140 unique genes identified across solid tumors such as head and neck, breast, lung, and liver cancers (Table S1). This curated gene set was then systematically applied to the TCGA-OV transcriptomic and clinical dataset to identify survival-associated candidates and to construct an ovarian cancer–specific prognostic model.

### 3.2 Identification of a Six-Gene Prognostic p-EMT Signature

We next evaluated the curated p-EMT gene set to identify candidates associated with overall survival. In the TCGA-OV cohort, univariate Cox regression identified ten genes significantly correlated with OS (*p* < 0.05), including ADAM9, ANXA8L1, FSTL3, HTRA1, PDPN, RABAC1, TGFB1, TPM4, TWIST1, and ZEB1 (Table 1). To derive a robust prognostic signature, these candidates were further subjected to LASSO regression, which selected six genes with non-zero coefficients—ADAM9, ANXA8L1, FSTL3, RABAC1, TPM4, and TWIST1—which constituted the final p-EMT signature for subsequent risk score construction and patient stratification.

**Table 1:** Hazard ratios and LASSO coefficients of OS-related p-EMT genes identified in the TCGA-OV cohort.

Gene	HR	*p*-Value	LASSO Coefficient
ADAM9	1.21	0.01	0.11293661
ANXA8L1	1.15	7.21 × 10^−4^	0.09513194
FSTL3	1.51	2.99 × 10^−3^	0.24237407
HTRA1	1.12	0.03	N/A
PDPN	1.10	0.05	N/A
RABAC1	1.21	0.01	0.13693357
TGFB1	1.13	0.02	N/A
TPM4	1.23	0.02	0.07911075
TWIST1	1.09	0.03	0.0280914
ZEB1	1.13	0.04	N/A

Note: This table summarizes the results of univariate Cox regression for candidate p-EMT genes, with corresponding hazard ratios (HR), *p*-values, and LASSO coefficients. Optimal lambda: 0.01427659. N/A: Not Applicable.

### 3.3 Performance of the p-EMT Risk Score

**Figure 2:**
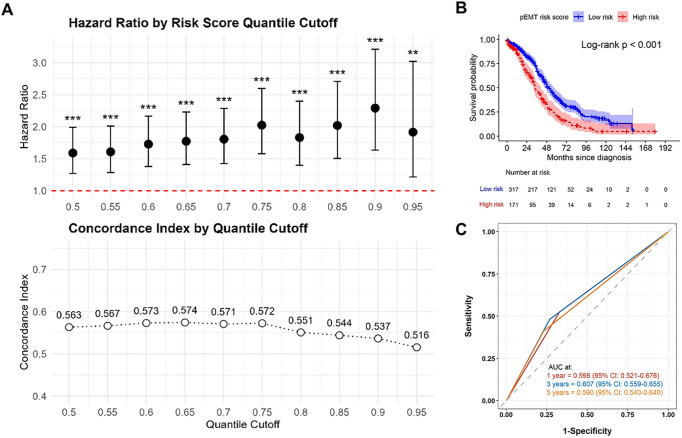
Prognostic performance of the p-EMT risk score in TCGA-OV dataset. (**A**) Hazard ratios (top panel) and concordance indices (C-index, bottom panel) across various quantile-based cutoffs of the p-EMT risk score. The 65th percentile was identified as the optimal threshold for prognostic stratification. (**B**) Kaplan–Meier survival curves comparing OS between high and low p-EMT risk score groups, stratified by the 65th percentile cutoff. Patients in the high p-EMT risk group exhibited significantly poorer survival outcomes (log-rank *p* < 0.001). (**C**) Time-dependent ROC curves illustrating the predictive accuracy of the p-EMT risk score for 1-, 3-, and 5-year overall survival (OS), with area under the curve (AUC) values of 0.598 (95% CI: 0.521–0.676), 0.607 (95% CI: 0.559–0.655), and 0.590 (95% CI: 0.540–0.640), respectively. **p < 0.01, ***p < 0.001.

To quantify the prognostic contribution of the six-gene p-EMT signature, we calculated a patient-specific p-EMT risk score as the weighted sum of the six gene expression levels using their LASSO-derived coefficients. To determine an appropriate cutoff for risk stratification, we evaluated a series of quantile thresholds ranging from the 50th to the 95th percentile. Although the 65th percentile yielded the highest concordance index (C-index; Fig. 2A) and was selected as the primary cutoff, the p-EMT score demonstrated consistent prognostic significance across this full range of thresholds, indicating that model performance was robust to alternative stratification strategies. Using the 65th-percentile cutoff, patients in the high-risk group exhibited significantly poorer overall survival (log-rank *p* < 0.001; Fig. 2B). Time-dependent ROC analysis showed modest predictive accuracy of the p-EMT risk score, with AUCs of 0.598 (95% CI: 0.521–0.676), 0.607 (95% CI: 0.559–0.655), and 0.590 (95% CI: 0.540–0.640) at 1-, 3-, and 5-year OS, respectively (Fig. 2C).

### 3.4 p-EMT Risk Score as an Independent Prognostic Factor in Ovarian Cancer

We next evaluated whether the p-EMT risk score functioned as an independent prognostic factor. High p-EMT scores were significantly enriched among patients who had died (Table 2), and mortality was likewise associated with older age and advanced FIGO stage (both *p* < 0.001). In univariate Cox regression, a high p-EMT risk score was strongly associated with increased mortality (HR = 1.77, 95% CI: 1.41–2.23, *p* < 0.001), consistent with the effects of age and FIGO stage (Table 3). Importantly, after adjustment for these clinical variables, the p-EMT risk score remained an independent predictor of overall survival (adjusted HR = 1.74, 95% CI: 1.38–2.19, *p* < 0.001; Table 4), indicating that its prognostic contribution is not explained by traditional clinicopathologic factors. The PH assumption was satisfied for the p-EMT risk score and stage, whereas age showed evidence of non-proportional hazards (*p* = 0.009) (Table S2).

**Table 2:** Clinical and demographic characteristics of TCGA-OV patients stratified by survival outcome.

Variable	Total (n = 488)	Survivor (n = 179)	Dead (n = 309)	*p*-Value
p-EMT risk score* median (IQR)	4.0 (3.9–4.2)	4.0 (3.9–4.2)	4.1 (3.9–4.3)	0.001
Low n (%)	317 (65.0)	131 (41.3)	186 (58.7)	0.004
High n (%)	171 (35.0)	48 (28.1)	123 (71.9)	N/A
Age, years median (IQR)	59.0 (51.0–69.0)	56.0 (49.0–65.0)	60.0 (53.0–70.0)	<0.001
<50 n (%)	96 (19.7)	52 (54.2)	44 (45.8)	<0.001
50~65 n (%)	223 (45.7)	80 (35.9)	143 (64.1)	N/A
>65 n (%)	169 (34.6)	47 (27.8)	122 (72.2)	N/A
Race	N/A	N/A	N/A	N/A
White n (%)	447 (91.6)	164 (36.7)	283 (63.3)	0.061
Asian n (%)	15 (3.1)	9 (60.0)	6 (40.0)	N/A
Other n (%)	26 (5.3)	6 (23.1)	20 (76.9)	N/A
FIGO stage	N/A	N/A	N/A	N/A
I n (%)	11 (2.3)	9 (81.8)	2 (18.2)	<0.001
II n (%)	25 (5.1)	17 (68.0)	8 (32.0)	N/A
III n (%)	377 (77.3)	135 (35.8)	242 (64.2)	N/A
IV n (%)	75 (15.4)	18 (24.0)	57 (76.0)	N/A
Tumor grade	N/A	N/A	N/A	N/A
1–2 n (%)	66 (13.5)	26 (39.4)	40 (60.6)	0.623
3–4 n (%)	422 (86.5)	153 (36.3)	269 (63.7)	N/A

Note: Abbreviations: IQR, interquartile range: 25th–75th percentile; p-EMT, partial epithelial-mesenchymal transition; FIGO, Federation of Gynecology and Obstetrics, N/A: Not Applicable. *: The Low and High level of p-EMT risk score was cut at 65th percentile in TCGA dataset: cut-off value = 4.14653160410553.

**Table 3:** Univariate cox regression for OS in the TCGA-OV cohort.

Variable	HR (95% CI)	*p* Value	Overall *p*-Value
p-EMT risk score*	N/A	N/A	<0.001
Low	ref	N/A	N/A
High	1.77 (1.41–2.23)	<0.001	N/A
Age, years (per 1-year increase)	1.03 (1.02–1.04)	<0.001	<0.001
Age, years (categorical)	N/A	N/A	<0.001
<50	ref	N/A	N/A
50~65	1.36 (0.97–1.91)	0.073	N/A
>65	2.08 (1.47–2.94)	<0.001	N/A
Race	N/A	N/A	0.074
White	ref	N/A	N/A
Asian	0.93 (0.41–2.09)	0.863	N/A
Other	1.70 (1.07–2.67)	0.023	N/A
FIGO stage	N/A	N/A	0.022
I-II	ref	N/A	N/A
III	2.04 (1.08–3.84)	0.028	N/A
IV	2.53 (1.29–4.96)	0.007	N/A
Tumor grade	N/A	N/A	0.183
1–2	ref	ref	N/A
3–4	1.25 (0.90–1.75)	0.183	N/A

Note: Abbreviations: HR, hazard ratio; CI, confidence interval; OS, overall survival; N/A: Not Applicable. *: The low and high level of p-EMT risk score was cut at 65th percentile in TCGA dataset: cut-off value = 4.14653160410.

**Table 4:** Multivariable cox regression for OS in the TCGA-OV cohort.

Variable	aHR (95% CI)	*p* Value	Overall *p*-Value
p-EMT risk score*	N/A	N/A	<0.001
Low	ref	N/A	N/A
High	1.74 (1.38–2.19)	<0.001	N/A
Age, years (per 1-year increase)	1.03 (1.02–1.04)	<0.001	<0.001
FIGO stage	N/A	N/A	0.025
I-II	ref	N/A	N/A
III	1.70 (0.90–3.22)	0.101	N/A
IV	2.30 (1.17–4.53)	0.016	N/A

Note: Abbreviations: p-EMT, partial epithelial-mesenchymal transition; FIGO, Federation of Gynecology and Obstetrics; aHR, adjusted hazard ratio; CI, confidence interval; N/A: Not Applicable. *: The low and high level of p-EMT risk score was cut at 65th percentile in TCGA dataset: cut-off value = 4.14653160410553.

### 3.5 Sensitivity Analysis

In sensitivity analyses, we fitted an extended Cox model including a time-dependent interaction term for age (age × log[time]) to account for the observed non-proportional hazards for age (Table S3). The time-dependent age term was statistically significant (adjusted HR = 0.86, *p* < 0.001), indicating that the effect of age varied over follow-up and tended to attenuate with increasing time. After incorporating this term, the direction of the association for the p-EMT risk score remained consistent (HR > 1), although its statistical significance was attenuated, while tumor stage remained significantly associated with the outcome.

### 3.6 Construction and Validation of the Prognostic Nomogram

**Figure 3:**
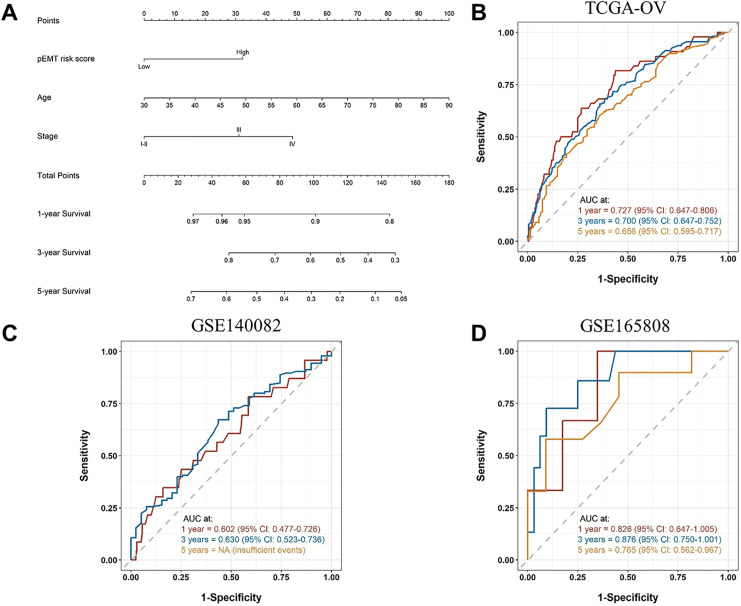
Construction and external validation of the p-EMT prognostic nomogram. (**A**) Nomogram developed from the TCGA-OV dataset incorporating the p-EMT risk score, patient age, and FIGO stage to estimate the probability of 1-, 3-, and 5-year OS. (**B**) Time-dependent ROC curves demonstrating the discriminative performance of the nomogram in the TCGA-OV cohort, with values of 0.727 (95% CI: 0.647–0.806), 0.700 (95% CI: 0.647–0.752), and 0.656 (95% CI: 0.595–0.717) for 1-, 3-, and 5-year OS, respectively. (**C**) External validation of the nomogram in the GSE140082 cohort demonstrated predictive AUCs of 0.602 (95% CI: 0.477–0.726) and 0.630(95% CI: 0.523–0.736) for 1-year and 3-year OS, respectively. Five-year OS data were not available in this dataset. (**D**) External validation of the nomogram in the GSE165808 cohort demonstrated predictive AUCs of 0.826(95% CI: 0.647–1.005), 0.876 (95% CI: 0.750–1.001), and 0.765 (95% CI: 0.562–0.967) for 1-, 3-, and 5-year OS, respectively.

To facilitate individualized risk assessment, we constructed a prognostic nomogram integrating the p-EMT risk score with age and FIGO stage (Fig. 3A). Each variable contributed a weighted point score in the multivariate Cox model, and the total score was used to estimate 1-, 3-, and 5-year OS probabilities. The integrated nomogram achieved AUCs of 0.727 (95% CI: 0.647–0.806), 0.700 (95% CI: 0.647–0.752), and 0.656 (95% CI: 0.595–0.717) at 1-, 3-, and 5-year OS, respectively, outperforming the p-EMT risk score alone (AUCs: 0.598, 0.607, 0.590; Figs. 2C and 3B). Calibration plots demonstrated close agreement between predicted and observed survival across all time points, supporting the model’s reliability and potential clinical utility (Fig. S1A–C).

### 3.7 External Validation of the p-EMT Prognostic Model in GSE140082

The prognostic model was externally validated in two independent cohort, GSE140082 (n = 380) and GSE165808 (n = 51), using the same p-EMT risk score formula and 65th-percentile cutoff derived from TCGA-OV (Table S4). Patients were successfully stratified into high- and low-risk groups, and the nomogram demonstrated comparable predictive performance to the training cohort. Time-dependent ROC analysis showed AUCs of 0.602 (95% CI: 0.477–0.726) and 0.630 (95% CI: 0.523–0.736) at 1- and 3-year OS, respectively, in GSE140082 (Fig. 3C), whereas 5-year OS estimation was not feasible due to limited follow-up. In the second validation cohort GSE165808, the model exhibited higher discriminative accuracy, with AUCs of 0.826 (95% CI: 0.647–1.005), 0.876 (95% CI: 0.750–1.001), and 0.765 (95% CI: 0.562–0.967) for 1-, 3-, and 5-year OS, respectively (Fig. 3D). Calibration plots confirmed close agreement between predicted and observed survival (Fig S1D,E). The full model achieved C-indices of 0.647 in GSE140082 and 0.742 in GSE165808 (Table S5), consistent with its performance in the TCGA training cohort. In addition to external validation, we further compared the prognostic performance of the p-EMT score with conventional clinical predictors. As summarized in Table S5, the clinical-only model (age and FIGO stage) demonstrated a C-index of 0.625 in TCGA, whereas the inclusion of the p-EMT risk score increased discrimination to 0.651. These findings indicate that the p-EMT signature provides incremental prognostic value beyond established clinical variables. Collectively, these results support the model’s robustness and generalizability across datasets.

## 4 Discussion

### 4.1 A Six-Gene p-EMT Signature Predicts Survival in Ovarian Cancer

In this study, we developed a six-gene transcriptomic signature derived from a curated pan-cancer p-EMT gene set and evaluated its association with OS in ovarian cancer. The p-EMT risk score stratified patients into groups with different survival outcomes and retained prognostic significance after adjustment for age and FIGO stage. When incorporated into a nomogram with clinical variables, the model demonstrated improved predictive performance, with time-dependent AUCs of 0.727, 0.700, and 0.656 at 1-, 3-, and 5-year OS, respectively. External validation in the GSE140082 and GSE165808 cohort supported the consistency of the model across datasets. These findings suggest that p-EMT–based molecular risk assessment may complement conventional clinicopathologic parameters and has the potential to aid individualized prognostic evaluation in ovarian cancer. These findings prompted further exploration of the functional implications of the six-gene signature.

### 4.2 Potential Functional Implications of the Six-Gene p-EMT Signature

p-EMT represents a dynamic intermediate state in which cancer cells acquire mesenchymal properties while retaining epithelial adhesion features. This hybrid phenotype increases cellular plasticity and has been closely linked to invasion, metastatic dissemination, and therapy resistance. Accumulating evidence supports p-EMT as a critical driver of cancer progression and a potential prognostic indicator [10]. In our study, the six-gene p-EMT signature—ADAM9, ANXA8L1, FSTL3, RABAC1, TPM4, and TWIST1—demonstrated a significant association with overall survival in ovarian cancer. TWIST1, a canonical EMT transcription factor, promotes mesenchymal reprogramming by suppressing epithelial gene expression [27]. ADAM9 contributes to extracellular matrix (ECM) remodeling and facilitates peritoneal dissemination [28]. FSTL3 regulates TGF-β signaling, a central axis in EMT activation and tumor progression [29]. TPM4 influences actin cytoskeletal organization and may enhance motility during transitional epithelial–mesenchymal states [30]. Although ANXA8L1 and RABAC1 are less characterized within EMT biology, their involvement in membrane trafficking and vesicle transport suggests potential roles in metastatic competence and phenotypic flexibility.

Together, these six genes participate in interconnected transcriptional, structural, and signaling processes that are characteristic of p-EMT biology. TWIST1 and FSTL3 support the maintenance of a hybrid epithelial/mesenchymal identity through transcriptional reprogramming and TGF-β pathway activation [31]. ADAM9 remodels the extracellular matrix and modifies cell–matrix interactions, facilitating partial epithelial disengagement without a complete mesenchymal shift [28]. Meanwhile, TPM4, RABAC1, and ANXA8L1 influence cytoskeletal architecture, membrane organization, and vesicle dynamics, collectively enhancing motility and facilitating phenotypic plasticity [32–34]. These coordinated mechanisms distinguish the biology of p-EMT from full EMT, wherein cells typically lose epithelial adhesion completely and adopt a terminal mesenchymal phenotype. These interconnected pathways may collectively provide a potential mechanistic basis for the prognostic relevance of the six-gene signature in ovarian cancer.

### 4.3 Advancing Prognostic Modeling by Targeting p-EMT in Ovarian Cancer

Several recent studies have leveraged EMT-related gene expression to construct prognostic models across different malignancies. For example, Li et al. developed a four-gene EMT signature composed of SNAI1, SMAD7, BMP2, and RGS3 to predict survival and immune microenvironment features in lung squamous cell carcinoma, achieving moderate performance with time-dependent AUCs of 0.587, 0.644, and 0.636 at 1-, 3-, and 5-year OS, respectively [35]. These models collectively underscore the biological relevance of EMT-associated transcriptional reprogramming in cancer outcomes. However, traditional EMT-based signatures largely quantify a fully mesenchymal state and therefore do not capture the intermediate, plastic cellular states characteristic of partial EMT (p-EMT). Accumulating evidence demonstrates that p-EMT—marked by the coexistence of epithelial adhesion features and mesenchymal motility programs—confers enhanced invasiveness, metastatic competence, and therapy resistance, properties not adequately represented by full-EMT markers. Pan-cancer analyses from EMTome further highlight the prognostic significance of hybrid E/M states in multiple tumor types, including ovarian cancer [36], yet these resources do not provide tumor-specific or clinically validated prognostic tools. In ovarian cancer, an EMT-based eleven-gene signature was recently proposed by Li et al., who additionally profiled EMT-related mutational features and reported widespread TP53 alterations with generally low mutation rates among other EMT regulators [26]. Notably, these genomic alterations were not incorporated into the predictive model, which remained exclusively transcriptomic. This illustrates a broader methodological limitation in EMT-oriented prognostic research: existing frameworks—including our own p-EMT–based model—primarily rely on gene-expression profiles while overlooking potentially informative genomic determinants. Future studies integrating p-EMT transcriptional signatures with mutational features—such as BRCA1/2 status, homologous recombination deficiency (HRD), or recurrent EMT-associated genetic alterations—may yield more comprehensive and clinically actionable prognostic systems. Incorporating additional genomic layers into p-EMT–based models may improve predictive accuracy and refine patient stratification in future studies.

## 5 Conclusion

In current study, we address a notable gap by developing a prognostic model specifically rooted in p-EMT biology, using a streamlined six-gene signature derived from literature-based pan-cancer evidence and optimized for ovarian cancer datasets. The integrated nomogram combining the p-EMT risk score with clinical variables demonstrated consistent performance across datasets. This p-EMT signature may serve as a molecular tool to refine risk stratification and inform clinical decision-making.

## 6 Limitation of the Study

Although the risk model demonstrated consistent prognostic performance, several limitations should be considered. Reliance on public datasets introduces potential biases, including batch effects and incomplete clinical annotations. Therapeutic information was particularly limited: the TCGA-OV cohort lacked detailed treatment-response data, while the GEO cohorts included only broad categorizations such as “bevacizumab” vs. “standard therapy,” preventing assessment of whether the p-EMT signature predicts differential benefit from specific therapeutic regimens. The incomplete and heterogeneous therapeutic information in TCGA-OV may also introduce residual confounding, as treatment effects could not be adequately accounted for when evaluating the prognostic contribution of the p-EMT score. Second, the study focused exclusively on transcriptomic measurements. Corresponding protein-level validation, spatial expression profiling, and mechanistic confirmation were not feasible due to data unavailability. As a result, the functional contribution of the six signature genes to p-EMT biology and ovarian cancer progression remains to be experimentally characterized. In addition, key clinical predictors such as postoperative residual disease, BRCA1/2 mutation, and immune infiltration metrics could not be incorporated into comparative analyses, because TCGA-OV does not provide standardized residual disease classification and lacks sufficiently harmonized treatment-response or immune-profiling annotations to evaluate their prognostic relevance. Although we evaluated a comprehensive range of quantile-based thresholds, the use of a percentile-derived cutoff—optimized at the 65th percentile—may limit immediate clinical generalizability, and future prospective studies will be needed to establish clinically standardized thresholds. Moreover, reduced predictive accuracy in the external validation cohort highlights the impact of inter-cohort variability. Taken together, these limitations highlight key areas for future work, including prospective cohort validation, functional and protein-level assays, integration of treatment-response data, and exploration of the model’s applicability across additional cancer types and clinical contexts.

## Supplementary Materials





## Data Availability

The datasets generated during and/or analysed during the current study are available from the corresponding author on reasonable request.

## Supplementary Materials

The supplementary material is available online at https://www.techscience.com/doi/10.32604/or.2026.074383/s1.
