# Supplementary material for: A Novel Partial EMT-Associated Transcriptomic Signature for Prognostic Stratification in Ovarian Cancer
Source: Oncol Res. 2026 Apr 22;34(5):27. doi: 10.32604/or.2026.074383 (PMC13126371; doi:10.32604/or.2026.074383)
Supplement: Supplementary file 1 [file OncolRes-34-74383-s001.docx]

**Table S1:** Summary of studies reporting p-EMT-related genes across various cancer types.

| **No.** | **Citation** | **Cancer Types** | **p-EMT Related Genes** |
| --- | --- | --- | --- |
| 1 | Puram, Sidharth V et al. [17] | Head and neck squamous cell carcinoma (HNSCC) | *SERPINE1,TGFBI,MMP10,LAMC2,P4HA2,PDPN,ITGA5,LAMA3,CDH13,TNC,MMP2,EMP3,INHBA,LAMB3,VIM,SEMA3C,PRKCDBP,ANXA5,DHRS7,ITGB1,ACTN1,CXCR7,ITGB6,IGFBP7,THBS1,PTHLH,TNFRSF6B,PDLIM7,CAV1,DKK3,COL17A1,LTBP1,COL5A2,COL1A1,FHL2,TIMP3,PLAU,LGALS1,PSMD2,CD63,HERPUD1,TPM1,SLC39A14,C1S,MMP1,EXT2,COL4A2,PRSS23,SLC7A8,SLC31A2,ARPC1B,APP,MFAP2,MPZL1,DFNA5,MT2A,MAGED2,ITGA6,FSTL1,TNFRSF12A,IL32,COPB2,PTK7,OCIAD2,TAX1BP3,SEC13,SERPINH1,TPM4,MYH9,ANXA8L1,PLOD2,GALNT2,LEPREL1,MAGED1,SLC38A5,FSTL3,CD99,F3,PSAP,NMRK1,FKBP9,DSG2,ECM1,HTRA1,SERINC1,CALU,TPST1,PLOD3,IGFBP3,FRMD6,CXCL14,SERPINE2,RABAC1,TMED9,NAGK,BMP1,ESYT1,STON2,TAGLN,GJA1* |
| 2 | Kisoda, Satoru et al. [13] | Head and neck squamous cell carcinoma (HNSCC) | *MMP10,CDH13,MMP3,TGFB1,P4HA2,PDPN,SNAI2,SERPINE1,ITGA5,LAMC2,LAMA3,LAMB3,INHBA,TNC* |
| 3 | Parikh, Anuraag S et al. [18] | Oral cavity squamous cell carcinoma (OCSCC) | *PDPN,LAMB3,LAMC2* |
| 4 | Sinha, Dona et al. [11] | Review paper | *ADAM17,ADAM9,CTNNB1,CDH1,CDH2,CLDN1,CSF1,FN1,FGG,KRT18,LAMB3,MMP1,NGF,PDPN,PLAT,SERPINE1,SNAI1,SNAI2,TGFB1,TGFB2,TWIST1,VEGFA,VIM,XIAP,TJP1,ZEB1,MET,ITGB1* |
| 5 | Aggarwal, Vaishali et al. [19] | Review paper | *S100A6,VEGFA,NFE2L2,L1CAM,CTSB,COL2A1,FN1,LAMC2,CD44* |
| 6 | Jolly, Mohit Kumar et al. [20] | Non-small cell lung cancer (NSCLC) | *GRHL2,miR-145,OVOL1* |
| 7 | Karaosmanoğlu, Oğuzhan et al. [21] | Hepatocellular carcinoma cells (HCC) | *FN1,COL2A1,FGG* |
| 8 | Kröger, Cornelia et al. [22] | Breast cancer | *ITGB4,CD44,SNAI1* |
| 9 | Sample, Reilly A et al. [23] | Review paper | *SNAI1,SNAI2,TWIST1,TWIST2,ZEB1,ZEB2* |
| 10 | Liao, Tsai-Tsen, and Muh-Hwa Yang. [12] | Review paper | *NFE2L2,NUMB,OVOL2,VCAM1,ITGB3,ITGAV,ZEB1,ITGB4,CD44* |

**Table S2:** Supremum Test for the Proportional Hazards Assumption.

| **Covariate** | **Maximum Absolute Value** | ***p*-value (Pr > MaxAbsVal)** |
| --- | --- | --- |
| p-EMT risk score | 1.10 | 0.146 |
| Age | 1.61 | 0.009 |
| FIGO stage | N/A. | N/A. |
| Stage III | 2.39 | 0.232 |
| Stage IV | 2.84 | 0.105 |

The proportional hazards assumption was evaluated using the supremum test based on scaled Schoenfeld residuals with 1,000 resampling iterations. A p-value < 0.05 indicates evidence against the proportional hazards assumption. N/A: Not Applicable.

**Table S3:** Sensitivity Analysis Using a Time-Varying Effect of Age in the Cox Model.

| **Covariate** | **HR (95% CI)** | ***p*-value** |
| --- | --- | --- |
| p-EMT risk score | 1.24 (0.97-1.58) | 0.088 |
| Age (per 1-year increase)* | 1.75 (1.65-1.85) | <0.001 |
| Age × log(time)* | 0.86 (0.85-0.87) | <0.001 |
| Stage III vs Stage I | 3.71 (1.71-8.05) | 0.001 |
| Stage IV vs Stage I | 4.74 (2.08-10.79) | <0.001 |

*A sensitivity analysis used an extended Cox model with a time-varying age effect (age × log(time)). HRs for age vary over time, whereas HRs for other covariates represent average effects.

**Table S4:** Comparison of Clinical and Molecular Characteristics Between TCGA-OV and GSE140082 Cohorts in Ovarian Cancer Patients.

|  | **TCGA (n=488)** | **GSE140082 (n=380)** | **TCGA vs. GSE140082 *P*-value** | **GSE165808 (n=51)** | **TCGA vs GSE165808 *P*-value** |
| --- | --- | --- | --- | --- | --- |
| p-EMT risk score median (IQR) | 4.0 (3.9-4.2) | 6.8 (6.4-7.2) | **<0.001** | 3.1 (2.9-3.4) | <0.001 |
| p-EMT risk score (categorical) * | N/A. | N/A. | N/A. | N/A. | N/A. |
| Low n (%) | 317 (65.0) | 247 (65.0) | 0.990 | 33 (64.7) | 0.971 |
| High n (%) | 171 (35.0) | 133 (35.0) | N/A. | 18 (35.3) | N/A. |
| Age, years median (IQR) | 59.0 (51.0-69.0) | 59.0 (49.5-66.0) | **0.042** | 55.0 (47.0-64.0) | 0.004 |
| Age, years (categorical) | N/A. | N/A. | 0.171 | N/A. | 0.053 |
| <50 n (%) | 96 (19.7) | 95 (25.0) | N/A. | 17 (33.3) | N/A. |
| 50~65 n (%) | 223 (45.7) | 162 (42.6) | N/A. | 22 (43.1) | N/A. |
| >65 n (%) | 169 (34.6) | 123 (32.4) | N/A. | 12 (23.5) | N/A. |
| FIGO stage | N/A. | N/A. | **0.009** | N/A. | 0.502 |
| I-II n (%) | 36 (7.4) | 51 (13.4) | N/A. | 5 (9.8) | N/A. |
| III n (%) | 377 (77.3) | 266 (70.0) | N/A. | 41 (80.4) | N/A. |
| IV n (%) | 75 (15.4) | 63 (16.6) | N/A. | 5 (9.8) | N/A. |
| Death n (%) | 309 (63.3) | 96 (25.3) | **<0.001** | 11 (21.6) | <0.001 |
| Follow-up, month median (IQR) | 34.2 (16.8-56.5) | 25.2 (20.2-31.0) | **<0.001** | 40.3 (23.0-55.5) | 0.122 |

*The Low and High level of pEMT risk score was cut at 65^th^ percentile in each dataset: cut-off value in TCGA = 4.15; cut-off value in GSE140082 = 7.08 and in GSE165808 = 3.25. Abbreviations: IQR, interquartile range:25^th^-75^th^ percentile; p-EMT, partial epithelial-mesenchymal transition; FIGO, Federation of Gynecology and Obstetrics; N/A: Not Applicable.

**Table S5:** Comparison of models based on different p-EMT risk score cut-offs and predictors.

| **Concordance/Model** | **p-EMT risk score -Continuous** | **p-EMT risk score -cut at 0.50** | **p-EMT risk score -cut at 0.65** |
| --- | --- | --- | --- |
| **TCGA (n=488)** |  | | |
| Model*, C-index (SE) | 0.651 (0.017) | 0.641 (0.018) | 0.651 (0.017) |
| Cov only*, C-index (SE) | 0.625 (0.018) | 0.625 (0.018) | 0.625 (0.018) |
| pEMT score, C-index (SE) | 0.591 (0.017) | 0.563 (0.016) | 0.574 (0.015) |
| **GSE140082** **(n=380)** |  | | |
| Model*, C-index (SE) | 0.611 (0.033) | 0.600(0.031) | 0.647 (0.03) |
| **GSE165808 (n=51)** |  | | |
| Model*, C-index (SE) | 0.697 (0.082) | 0.699(0.074) | 0.742 (0.069) |

*Model includes age, stage, and p-EMT risk score. Cov only includes age and stage. Abbreviations: C-index: concordance index; SE: standard error.


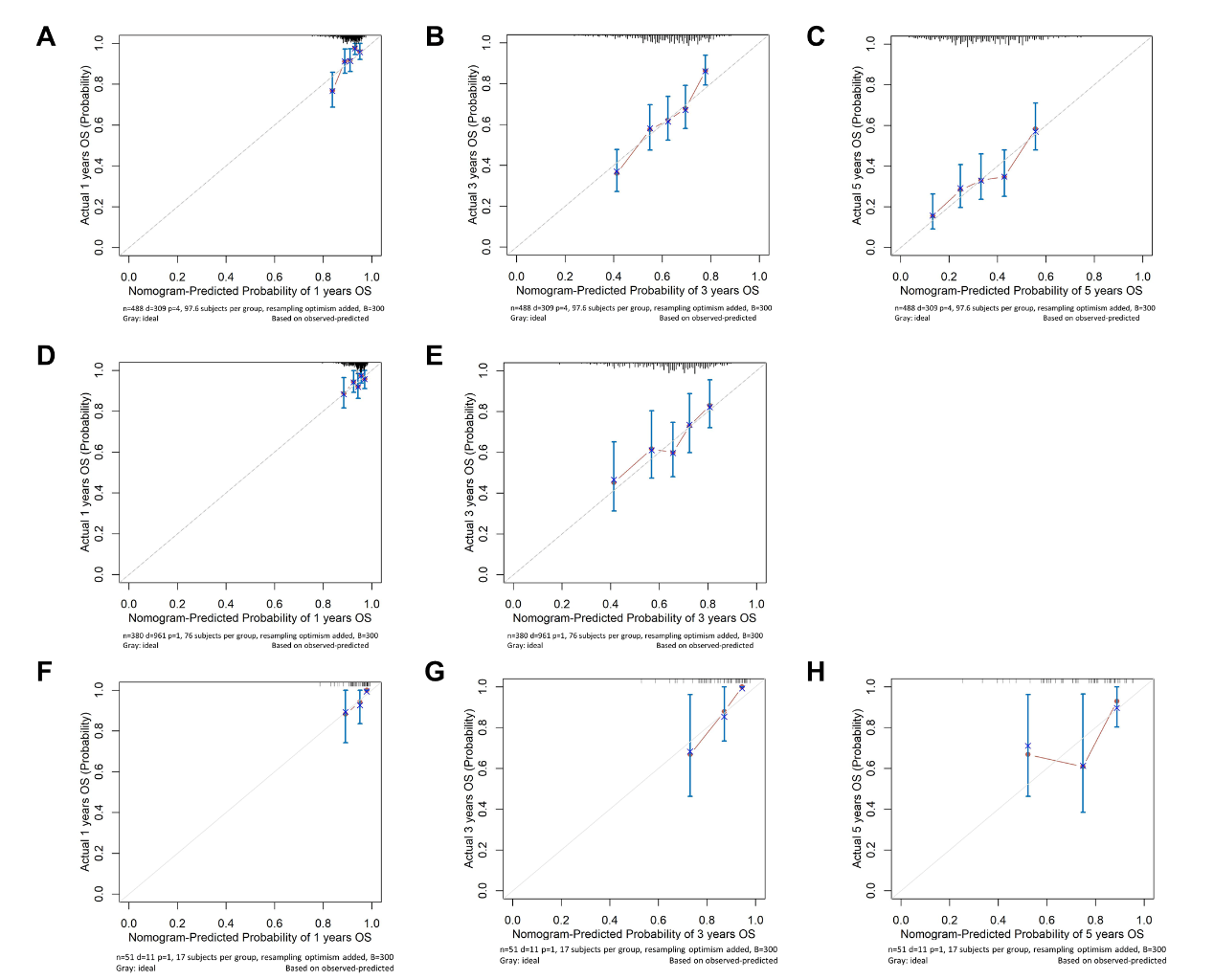


**Figure S1.** **Calibration plots evaluating the predictive accuracy of the nomogram for overall survival (OS) in the TCGA-OV, GSE140082 and GSE165808.** (A–C) The plots compare the predicted and observed OS probabilities at 1, 3, and 5 years in the TCGA-OV dataset. The dashed gray line represents the ideal prediction. (D–E) The plots compare the predicted and observed OS probabilities at 1, 3, and 5 years in the GSE140082 dataset. The dashed gray line represents the ideal prediction. (F–H) The plots compare the predicted and observed OS probabilities at 1, 3, and 5 years in the GSE165808 dataset. The dashed gray line represents the ideal prediction.
