# Supplementary figures and images for: A Novel Partial EMT-Associated Transcriptomic Signature for Prognostic Stratification in Ovarian Cancer
Source: Oncol Res. 2026 Apr 22;34(5):27. doi: 10.32604/or.2026.074383 (PMC13126371; doi:10.32604/or.2026.074383)

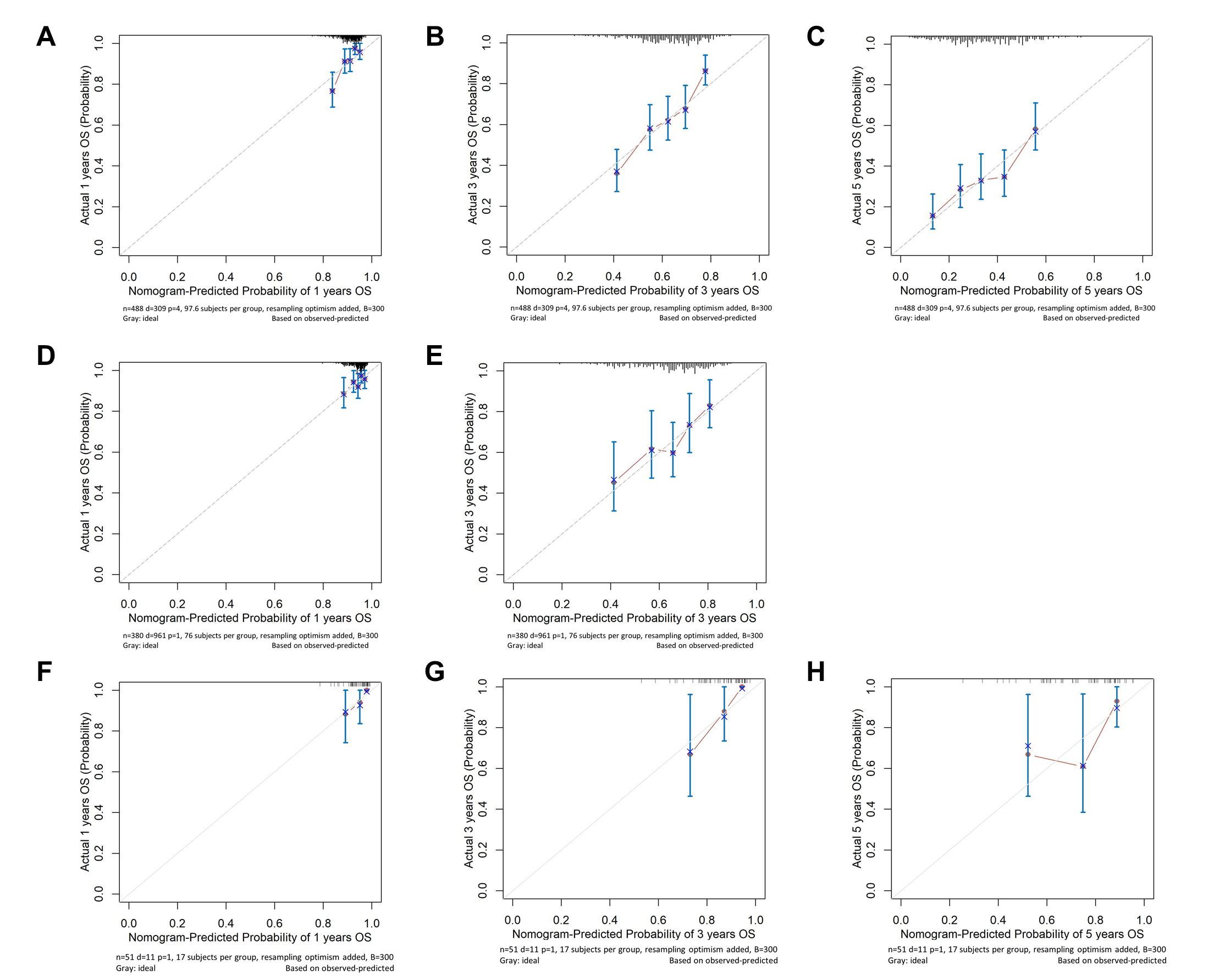

Supplement: Supplementary file 2 [file OncolRes-34-74383-s002.tif]
